# Improving Existing Segmentators Performance with Zero-Shot Segmentators

**DOI:** 10.3390/e25111502

**Published:** 2023-10-30

**Authors:** Loris Nanni, Daniel Fusaro, Carlo Fantozzi, Alberto Pretto

**Affiliations:** Department of Information Engineering, University of Padova, 35122 Padua, Italy; fusarodani@dei.unipd.it (D.F.); carlo.fantozzi@unipd.it (C.F.); alberto.pretto@unipd.it (A.P.)

**Keywords:** segmentation, deep learning, ensemble, zero-shot segmentator

## Abstract

This paper explores the potential of using the SAM (Segment-Anything Model) segmentator to enhance the segmentation capability of known methods. SAM is a promptable segmentation system that offers zero-shot generalization to unfamiliar objects and images, eliminating the need for additional training. The open-source nature of SAM allows for easy access and implementation. In our experiments, we aim to improve the segmentation performance by providing SAM with checkpoints extracted from the masks produced by mainstream segmentators, and then merging the segmentation masks provided by these two networks. We examine the “oracle” method (as upper bound baseline performance), where segmentation masks are inferred only by SAM with checkpoints extracted from the ground truth. One of the main contributions of this work is the combination (*fusion*) of the logit segmentation masks produced by the SAM model with the ones provided by specialized segmentation models such as DeepLabv3+ and PVTv2. This combination allows for a consistent improvement in segmentation performance in most of the tested datasets. We exhaustively tested our approach on seven heterogeneous public datasets, obtaining state-of-the-art results in two of them (CAMO and Butterfly) with respect to the current best-performing method with a combination of an ensemble of mainstream segmentator transformers and the SAM segmentator. The results of our study provide valuable insights into the potential of incorporating the SAM segmentator into existing segmentation techniques. We release with this paper the open-source implementation of our method.

## 1. Introduction

Semantic image segmentation aims to assign each pixel in an image to a specific object class, enabling a more fine-grained understanding of visual content. Over the years, deep learning models have significantly advanced the field, demonstrating remarkable achievements in accurately segmenting objects within complex scenes. Among these models, DeepLabv3+ [1] has garnered substantial attention due to its ability to capture detailed object boundaries while maintaining computational efficiency.

However, a fundamental challenge faced by DeepLabv3+ and other mainstream models lies in their ability to generalize to unfamiliar objects and images. In these cases, these models often struggle to produce accurate segmentations, as they lack the necessary knowledge to recognize and segment such objects effectively. This limitation restricts the practical deployment of segmentation models in real-world scenarios, where encountering novel objects is a common occurrence. Recently, two cutting-edge promptable segmentation systems, SAM [2] and SEEM [3], have been proposed. They offer zero-shot generalization capabilities to unfamiliar objects and images without requiring additional training. SAM and SEEM leverage the powerful concept of prompting, which allows users to input specific instructions or hints to guide the model’s behavior. We propose to leverage SAM and SEEM alongside DeepLabv3+ to extend their segmentation accuracy when dealing with novel, unconventional objects belonging to known classes. Although DeepLabv3+ may not currently represent the state-of-the-art (SOTA) in semantic segmentation (the current SOTA is obtained by transformers like [4]), it remains a highly popular and widely used segmentator, serving as a valuable baseline for evaluating the performance of the SAM and SEEM models.

Broadly speaking, our approach involves extracting checkpoints from the segmentation masks produced by mainstream models such as DeepLabv3+ and utilizing them as prompts for zero-shot segmentators such as SAM and SEEM. We see two fundamental reasons why zero-shot segmentators can improve the quality of segmentation with respect to mainstream models, including specialized models trained for a specific task.

1. Zero-shot segmentators are trained on more images (billions of them, in the case of SAM) than mainstream and specialized models. This fact may allow them to beat the SOTA in some circumstances.
2. Differences in the architecture and training set of the zero-shot segmentator with respect to the mainstream and/or specialized models are a source of diversity, which is the ideal prerequisite for a strong ensemble. The literature shows that ensemble models have repeatedly improved the SOTA on several tasks. Indeed, in this paper our approach allows us to improve the SOTA with an ensemble, as better explained later.

In addition, zero-shot segmentators are trained on images belonging to several different classes. As a consequence, in principle, our approach should provide acceptable performance, even when not beating the SOTA, in many scenarios, particularly those involving unfamiliar objects, and without the need of retraining/fine-tuning the models, which is a benefit in real-world situations.

In this paper, we present a comprehensive analysis of the proposed approach, assessing its impact on segmentation quality, generalization to unfamiliar objects, and computational efficiency. As a noteworthy contribution, we propose to fuse the logit segmentation mask provided by SAM with the logit mask provided by the segmentation model exploited to extract the checkpoints. This fusion configuration produces consistently better results than the base models. To provide a baseline for comparison, we also investigate the method of using checkpoints extracted from ground truth segmentation masks, which we refer to as the “oracle” method. We carry out experiments on seven heterogeneous benchmark datasets, comparing the performance of DeepLabv3+ with and without SAM and SEEM integration. The best performance is obtained by combining SAM with DeepLabv3+ using the proposed mask fusion strategy. Only for some datasets, due to computational problems, we also run an ensemble of PVTv2 transformers [4], whose fusion with SAM obtains the new SOTA for CAMO and Butterfly datasets. Additionally, we evaluate the effectiveness of the “oracle” method to provide insights into the potential benefits of leveraging ground truth information.

The contributions of this paper are the following: (i) we empirically prove that a careful combination of a specialized segmentation model with a zero-shot segmentator like SAM can generally improve the segmentation results at no cost, obtaining in some cases SOTA results; (ii) we report an extensive performance evaluation on seven heterogeneous datasets that support our claims, where we used two different segmentation models and two alternative zero-shot segmentators; (iii) among other outcomes, our experiments demonstrate how the fusion of a zero-shot segmentator with a specialized model can rival and even surpass the fusion of two state-of-the-art specialized models, with the difference that in the latter case, a double train is required in the training subset of the target dataset; (iv) we release with this paper the open-source implementation of our method, freely available at https://github.com/LorisNanni (accessed on 23 July 2023).

The remainder of the paper is organized as follows. Section 2 provides an overview of related work in the field of semantic segmentation and zero-shot learning. Section 3 outlines the methodology, including the architecture of DeepLabv3+, the promptable segmentation systems SAM and SEEM, and the proposed integration approach. Section 4 presents the experimental setup. Section 5.1 displays the results of the different methods and a discussion of these results is carried out. Section 6 concludes the paper.

## 2. Related Work

The related work section provides an overview of the existing literature in the field of semantic segmentation, focusing on three key aspects: deep learning-based segmentation methods, zero-shot learning in segmentation, and combining continuous outputs of different classifiers to improve performance. These areas of research have contributed significantly to the advancement of the field, addressing challenges related to accurate and efficient segmentation.

### 2.1. Deep Learning-Based Segmentation Methods

Deep learning-based segmentation methods have emerged as powerful techniques for pixel-level object classification in images. These methods capitalize on the capabilities of deep neural networks to capture intricate details and contextual information, allowing the accurate segmentation of objects within complex scenes. U-Net [5] is a pioneering architecture for image segmentation. It comprises a contracting path, which captures context information, and an expansive path, which refines spatial details. U-Net’s skip connections facilitate the fusion of feature maps from different resolutions, aiding in accurate pixel-wise predictions and enabling its successful application in medical image segmentation tasks. SegNet [6] is another popular semantic segmentation model, designed to balance segmentation accuracy with computational efficiency. It uses an encoder-decoder architecture with a trainable decoder for pixel-wise predictions. SegNet is known for its compact structure, which makes it suitable for real-time applications in various domains. DeepLabv3+ [1] is an extension of the DeepLab family, featuring Atrous Spatial Pyramid Pooling (ASPP) and encoder-decoder modules. ASPP captures multi-scale contextual information, while the encoder-decoder module refines segmentation boundaries. DeepLabv3+ is widely recognized for its strong performance in large-scale and real-world segmentation tasks. HRNet [7] is a recent model that retains high-resolution representations of the input throughout the network, thus capturing fine-grained details in addition to global contextual information. This is achieved by maintaining parallel sub-networks that process the input image at different scales, allowing the network to effectively integrate features from various levels of granularity. HRNet has demonstrated notable performance on various benchmarks for semantic segmentation. By preserving high-resolution details, it excels at distinguishing fine-grained object boundaries and accurately capturing object shapes, even in challenging scenarios.

Vision transformers [8] introduced the transformer architecture to image classification tasks and have since been adapted to image segmentation. ViT models process images in a patch-based manner and employ self-attention mechanisms to capture long-range dependencies. Despite being designed for classification, they have also shown promising results in semantic segmentation. Many transformer-based segmentation methods have been proposed in recent years. For instance, the Object-Contextual Representation (OCR) model [9] exhibits two transformer-based branches: one branch for object recognition and another for contextual understanding. By considering the relationships between objects and their context, OCR can distinguish between objects with similar features, leading to more accurate segmentation results, e.g., for objects that are partially occluded or tightly packed together. The SegFormer model [10] brings transformer-based models originally developed for natural language processing to the domain of image segmentation. Transformers are adopted to capture long-range dependencies and contextual relationships. Pyramid Vision Transformer (PVT) v2 [4] is an extension of the ViT architecture designed to improve efficiency and scalability. PVTv2 combines the advantages of both convolutional and transformer models, leveraging multi-scale representations through pyramid structures. This allows PVTv2 to achieve competitive performance in various vision tasks, including semantic segmentation.

It should also be mentioned that several works improve the performance of the models mentioned above by considering additional information (for instance, the semantic relations between pixels across different images [11] or the statistical distribution of pixels in each class [12]) while building the models themselves.

### 2.2. Zero-Shot Learning in Segmentation

Zero-shot learning plays a crucial role in segmentation tasks, especially when faced with unfamiliar objects during inference. Traditional segmentation models often struggle to generalize to novel or unseen object classes, as they lack the necessary knowledge to effectively recognize and segment such objects. SAM, an image segmentation model [2], stands out as an innovative approach to promptable image segmentation. Trained on a vast dataset comprising more than one billion segmentation masks, SAM exhibits impressive zero-shot generalization capabilities. It excels in producing high-quality masks even from a single foreground point. The HQ-SAM model [13] is an extension of SAM that introduces a learnable high-quality output token. This addition improves the effectiveness of the model in various segmentation domains, resulting in improved performance. Although SAM may not provide high-quality segmentation directly for medical image data [14,15,16,17], its masks, features, and stability scores can be utilized to improve medical image segmentation models. SAMAug [18] is a method that leverages SAM to augment image input for commonly-used medical image segmentation models, boosting the performance of both CNN and transformer models. Another work focusing on performance for medical images is [19], which modifies only the SAM conditioning encoder part (mask or set of points). A new encoder is placed at the beginning, trained using the gradients provided from the frozen SAM subsequent architecture, and SOTA levels are reached in many datasets.

### 2.3. Combining Continuous Outputs

Several approaches have been proposed to combine continuous outputs in the field of image segmentation. For a comprehensive list of combined approaches, please refer to [20,21].

One commonly used technique is the weighted rule, which aggregates the predicted probability maps or logits from multiple models or methods. This rule has shown effectiveness in various segmentation tasks. Many fusion-based methods have been proposed in recent years; here, we describe two to elucidate how they work. In [22], a multi-label classifier system based on CNN and LSTM networks for ATC prediction is employed. A 1D feature vector from a compound is extracted and transformed into 2D matrices. A CNN model is trained using these matrices to extract a set of new features. In parallel, an LSTM model is trained on the original 1D vector to extract complementary features. These features are then fed into two general-purpose classifiers specifically designed for multi-label classification. Finally, the outputs of the classifiers are fused using the average rule to generate the final prediction results. The average rule is a weighted rule in which each classifier has the same weight.

Another study [23] focuses on the classification of pedestrians using deep learning techniques with data from a monocular camera and a 3D LiDAR sensor. The outputs from individual different CNNs are combined through learning and non-learning (average, minimum, maximum, and normalized-product) approaches. From the experimental results, fusion strategies obtain better results compared to individual CNNs. In particular, the average rule obtains promising results.

## 3. Methodology

Motivated by the challenges of the datasets used and the capabilities of the zero-shot semantic segmentation methods SAM and SEEM, we study how these methods can improve the performance of mainstream segmentation approaches on such datasets. In this section, we first illustrate the architecture of all the segmentators we use. Then, we describe the methods we consider to generate prompts for the zero-shot segmentators.

### 3.1. DeepLabv3+ Architecture

DeepLabv3+ is a popular semantic segmentation model that has demonstrated impressive performance in accurately segmenting objects within images. At its core, DeepLabv3+ utilizes a Fully Convolutional Network (FCN) structure, enabling end-to-end training and inference on arbitrary-sized images. The network architecture consists of an encoder-decoder structure that leverages atrous convolutions and Atrous Spatial Pyramid Pooling (ASPP) to capture multi-scale contextual information. DeepLabv3+ also introduces a skip connection from the encoder to the decoder module to incorporate low-level details from early layers of the network. This skip connection helps to refine the segmentation boundaries and improve the localization accuracy of the segmented objects.

Overall, DeepLabv3+ combines the strengths of atrous convolutions, ASPP, and skip connections to achieve SOTA segmentation results. Its architecture makes it possible to capture detailed object boundaries while maintaining computational efficiency, making it an excellent candidate for integration with the SAM segmentator.

### 3.2. Pyramid Vision Transformer Architecture

The Pyramid Vision Transformer (PVT) [4] stands as a transformer network devoid of convolutions. Its core concept revolves around acquiring high-resolution representations from finely-detailed input. The network’s depth is paired with a progressively narrowing pyramid, enabling a reduction in computational burden. Additionally, to further curtail computational overhead, the system incorporates a Spatial-Reduction Attention (SRA) layer. Each PVT network is trained for 50 epochs with a batch size of 8. AdamW is used as the optimizer. In this work, we use an ensemble of six networks, combined by average rule, constructed as follows:

- we apply two different data augmentation, defined in [24]: DA1, a base data augmentation consisting in horizontal and vertical flip, 90° rotation; DA2, which applies a rich set of diverse operations to derive new images from the original ones. These operations encompass shadowing, color mapping, vertical or horizontal flipping, and others.
- we apply three different learning strategies: learning rate of $1\times 10^{-4}$; learning rate of $5\times 10^{-4}$ decaying to $5\times 10^{-5}$ after 10 epochs; learning rate of $5\times 10^{-5}$ decaying to $5\times 10^{-6}$ after 15 epochs and to $5\times 10^{-6}$ after 30 epochs.

The six networks are obtained by coupling the two DA methods with the three strategies to determine the learning rate.

### 3.3. SAM Architecture

SAM (Segment-Anything Model) [2] is a SOTA vision foundation model specifically designed for promptable image segmentation. It has been trained on the extensive SA-1B dataset, which includes 11 million images and more than 1 billion masks, making it the largest segmentation dataset to date. This vast training set enables SAM to demonstrate exceptional zero-shot generalization capabilities when applied to new data. SAM has proven its ability to generate high-quality masks even with just a single foreground point and has shown robust generalization across various downstream tasks, such as edge detection, object proposal generation, and instance segmentation.

The SAM model consists of three main components: an image encoder, a flexible prompt encoder, and a fast mask decoder. The image encoder utilizes a Vision Transformer (ViT) backbone to process high-resolution 1024 × 1024 images and generate a 64 × 64 image embedding. The prompt encoder handles both sparse prompts (e.g., points, boxes, text) and dense prompts (e.g., masks) by converting them into *c*-dimensional tokens. Finally, the lightweight mask decoder combines the image and prompt embeddings to produce segmentation masks in real-time. This design allows SAM to efficiently handle diverse prompts with minimal computational overhead.

In our study, we evaluated two versions of the SAM model: ViT-Huge (ViT-H) and ViT-Large (ViT-L). The models vary in the complexity of the input image vision transformer-based encoder, with the former model having 632 M parameters and the latter having 307 M parameters.

### 3.4. SEEM Architecture

SEEM is a promptable, interactive model for Segmenting Everything Everywhere all at once in an image, as described in [3]. The system aims to predict masks and semantic concepts based on the interactions between the input image and multi-modal prompts. To do this, it encodes points, masks, text, boxes, or even a similar referred region of another image in the same joint visual-semantic space.

SEEM employs a generic encoder-decoder architecture, which consists of an image encoder that extracts features from the input image, which are then used by the SEEM decoder to predict masks and semantic concepts. Learnable queries interact with visual, text, and memory prompts through a self-attention mechanism.

It is important to note that the panoptic and interactive segmentation parts of the SEEM model are trained with COCO2017 [25] with panoptic segmentation annotations.

### 3.5. Checkpoint Engineering

**Figure 1:**
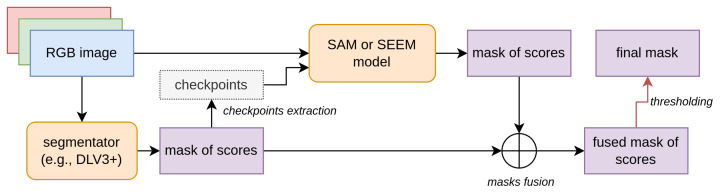
Visual scheme of the architecture of our system. An input RGB image is firstly processed by a segmentator, e.g., DeepLabv3+. It produces in output a segmentation mask of scores. From this mask we extract checkpoints. These checkpoints will be the input, along with the original RGB image, of a zero-shot segmentator such as SAM or SEEM. This model will in turn produce another segmentation mask of scores. Through the fusion process we condensate the two segmentation masks of scores of the first segmentator and the zero-shot segmentator. The final segmentation mask is obtained by thresholding the fused mask.

We devised several methods to generate checkpoints (prompts). The goal of checkpoint engineering is to investigate whether a specific prompt generation method can enhance the performance of a prompt-based segmentator. Our system (see Figure 1 for an illustration of the architecture) takes as input an image along with its segmentation mask. The segmentation mask specifically identifies a particular class of objects by separating them from the remaining pixels, which represent the background. Note that in this work we are dealing with only two classes of data (background and foreground). This segmentation mask can be either the ground truth mask or the output of a segmentation model. Throughout this paper, we will refer to this segmentation mask as the “source image mask.” It is important to note that source image masks may be composed of several regions (“blobs”), disconnected from each other, masking several portions of the image belonging to the same class of interest.

We devised four different methods to generate checkpoints starting from a source image mask, which we refer to as “A”, “B”, “C”, and “D”.

A selects the average coordinates of the blob as the checkpoint. While simple and straightforward, a drawback of this method is that checkpoints may occasionally fall outside the blob region.
B determines the center of mass of the blob as the checkpoint. It is similar to Method A and is relatively simple, but we observed that the extracted checkpoints are less likely to lie outside the blob region.
C randomly selects a point within the blob region as the checkpoint. The primary advantage of this method is its simplicity and efficiency. By randomly selecting a point within the blob, a diverse range of checkpoints can be generated.
D enables the selection of multiple checkpoints within the blob region. Initially, a grid is created with uniform sampling steps of size *b* in both the x and the y directions. Checkpoints are chosen from the grid if they fall within the blob region. We also applied a modified version of this method that considers eroded (smaller) masks. In Table 2, this modified version is referred to as “bm” (border mode). Erosion is a morphological image processing technique used to reduce the boundaries of objects in a segmentation mask. It works by applying a predefined kernel, in our case an elliptical-shaped kernel with a size of 10 × 10 pixels, to the mask. The kernel slides through the image, and for each position, if all the pixels covered by the kernel are part of the object (i.e., white), the central pixel of the kernel is set to belong to the output eroded mask. Otherwise, it is set to background (i.e., black). This process effectively erodes the boundaries of the objects in the mask, making them smaller and removing noise or irregularities around the edges. In certain cases, this method (both eroded and not) may fail to find any checkpoints inside certain blobs. To address this, we implemented a fallback strategy: the grid of checkpoints is shifted horizontally and then vertically, continuing this process while no part of the grid overlaps with the segmentation mask. The pseudo-code for Method D, including the fallback strategy, is provided in Algorithm 1.

**Algorithm 1** Method D with mask erosion and fallback strategy.

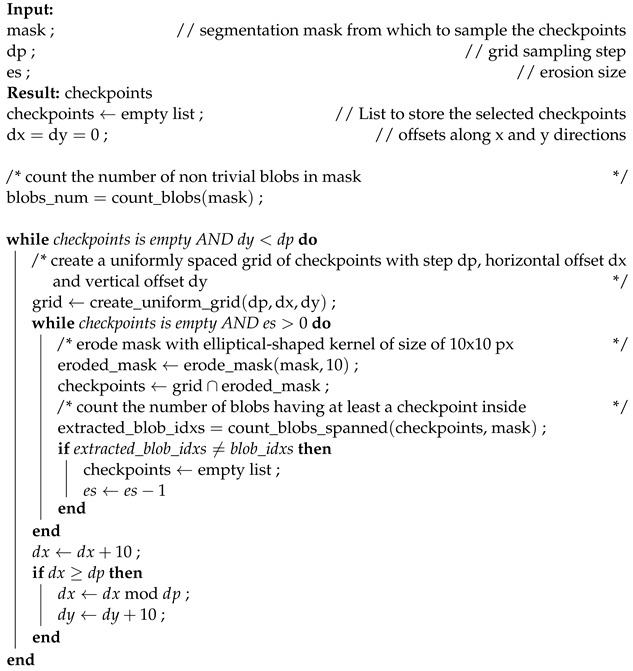



**Figure 2:**
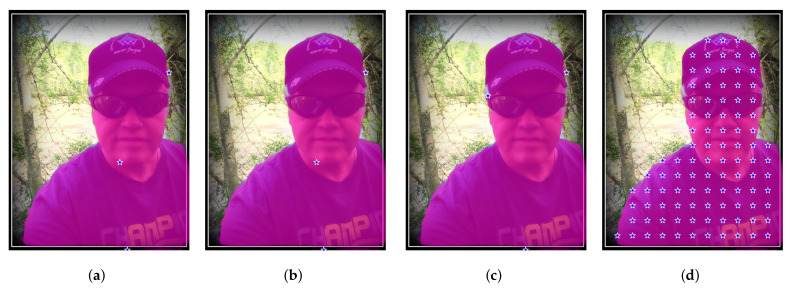
An example from the Portrait dataset showing the checkpoints extracted by the four methods we consider in this paper. The mask overlaid in orange is provided by DeepLabv3+ and, of course, is the same in the four cases shown. (**a**) Method A places a checkpoint at the average coordinates of each blob. (**b**) Method B puts a checkpoint at the center of mass of each blob. (**c**) Method C randomly selects a point within each blob. (**d**) Method D provides a grid of checkpoints.

An example of the checkpoints produced by the four methods is reported in Figure 2.

## 4. Experimental Setup

### 4.1. Datasets

In our experiments, we employed seven different datasets to evaluate the performance of our segmentation methods: CAMO, Portrait, Locust-mini, VinDr-RibCXR, SKIN, Butterfly, and a subset of COCO2017. Each dataset offers unique characteristics and challenges, which ensures a comprehensive evaluation. For each dataset, we use split training tests as reported in the literature, except for COCO2017 (please refer to the description).

The *CAMO* dataset [26] consists of images with diverse natural scenes containing objects of interest camouflaged in the background. It encompasses various challenging scenarios, such as objects with complex textures and occlusions, making it suitable for evaluating segmentation performance in real-world scenarios. The dataset contains a total of 1250 images, of which 1000 were used for training and 250 for testing.

The *Portrait* dataset [27] focuses specifically on portrait images of humans. It is designed to evaluate segmentation performance in the context of portrait photography, considering factors such as facial features, skin tones, and background elements. This dataset includes 1447 images for training and 289 images for validation; it can be accessed on https://github.com/HYOJINPARK/ExtPortraitSeg (accessed on 23 July 2023).

The *Locust-mini* dataset [28] contains a collection of 874 images in the training set and 120 test images featuring camouflaged locusts and grasshoppers on various backgrounds. This dataset poses unique challenges due to the complex color patterns and textures of the insects, making it suitable for evaluating segmentation performance in the context of camouflage detection.

The *VinDr-RibCXR* dataset [29] comprises chest X-ray images to detect and segment rib structures. Although it is intended primarily for rib segmentation, we utilized this dataset to evaluate the generalization capability of our proposed methods to medical imaging tasks. This dataset includes a training set of 196 images and a test set of 49 images.

The *SKIN* dataset is a collection of many skin segmentation datasets. A brief description of each is given in Table 1; for an in-depth analysis, please refer to [30].

The *Butterfly* dataset [31] is a public dataset (http://www.josiahwang.com/dataset/leedsbutterfly/) (accessed on 23 July 2023) for butterfly identification. For a fair comparison with previous results, we used the same testing protocol proposed by the authors of the dataset, that is, a four-fold cross-validation; each fold includes 208 test images and 624 training images. In this dataset we use resized images of size 513 × 513.

The *COCO 2017 Panoptic Image Segmentation* dataset [25] is a comprehensive collection of images with pixel-level annotations that classify objects into categories, providing both object instance masks and semantic segmentation labels. We used the COCO 2017 Training and Validation version, and used the validation part for testing. The dataset provides many different supercategories and categories, but we selected the supercategory “animal” and extracted all the images from the train/validation folders in which the supercategory label was present. In this way we produced a binary ground truth (background/animal) to train/test the different methods. The training folder contains 23,977 samples and the test folder contains 1016 samples. We refer to this dataset as *COCO_animals*.

### 4.2. Performance Metrics

To assess the segmentation performance, we employed two commonly used metrics: Intersection over Union (IoU) and Dice similarity coefficient (Dice). For the CAMO dataset we also computed the Mean Average Error (MAE), weighted F-measure, and E-measure, since many papers that segment that dataset also report these performance indicators.

IoU, which was introduced in [32], is defined as

(1) $$IoU\left(P,T\right)=\frac{P\cap T}{P\cup T},$$

where *P* is the predicted segmentation mask, *T* is the ground-truth mask, and the cardinality is the number of pixels. An IoU of 1 corresponds to a perfect prediction, that is, a pixel-perfect overlap between the predicted segmentation mask and the ground truth.

The Dice coefficient [33] is defined as

(2) $$Dice\left(P,T\right)=\frac{2P\cap T}{P+T}$$

and it measures the overlap between the predicted segmentation mask and the ground truth mask.

The Mean Absolute Error (MAE) metric [34] for 2D image semantic segmentation is a measure of the average absolute difference between the predicted segmentation masks and the ground-truth masks at the pixel level. It is defined as

(3) $$MAE\left(P,T\right)=\frac{\sum_{i=1}^{n}P_{i}-T_{i}}{n},$$

where *n* is the number of pixels of an image, and with $X_{i}$ we indicate the *i*-th pixel of image *X*. It quantifies the accuracy of the segmentation model by calculating the average pixel-wise absolute difference between the predicted and true masks for each class in the image. A lower MAE value indicates a better-performing segmentation model with higher accuracy in predicting the correct segmentation boundaries and class labels. MAE is commonly used to evaluate the performance of image segmentation models and to compare different approaches in the field of computer vision.

The weighted F-measure [35] is used to capture the relationship between precision and recall. This means that the F-measure considers the imbalance between classes and provides a more comprehensive evaluation of the segmentation model’s performance on different categories. A higher weighted F-measure indicates better overall segmentation accuracy, considering the varying class proportions. We use the weights suggested by the authors of CAMO.

The E-measure [36], also known as the Enhanced Dice Coefficient, is a performance metric used in binary semantic segmentation tasks to evaluate the accuracy of the predictions. It is an extension of the Dice coefficient and incorporates an additional term to penalize false positives and false negatives differently. This adjustment provides a more balanced evaluation, especially in cases of class imbalance, where the standard Dice coefficient might be biased towards the majority class. A higher E-measure value indicates better segmentation accuracy, considering both precision and recall of the predictions.

### 4.3. Baseline Extraction

The baseline performance in our experiments is established by evaluating the results of the DeepLabv3+ model, which was trained end-to-end on each of the datasets in this study. In addition, the PVTv2 segmentator ensemble is applied to the CAMO dataset.

For our experiments, we employed a DeepLabv3+ model with ResNet101 as the backbone architecture. The model was not trained from scratch. We started the training process from pre-trained weights on the Pascal VOC2012 Aug dataset [37], which consists of 513 × 513 RGB images from various categories (airplanes, buses, cars, trains, persons, horses, and more) of the original Pascal VOC2012 dataset augmented with extra annotations.

The hyperparameters for the training phase (DeepLabv3+) were as follows: an initial learning rate of 0.01, a total of 10 epochs for training, a momentum value of 0.9, L2 regularization with a coefficient of 0.005, a learning rate drop period of 5 epochs, a learning rate drop factor of 0.2, shuffling of training images at every epoch, and the adoption of the SGD (Stochastic Gradient Descent) optimizer. To increase the diversity and generalization capability of the model, data augmentation techniques were employed. Three operations, namely horizontal flip, vertical flip, and 90° rotation, were applied to augment the training set. These augmentation operations create additional variations of the training samples, thereby improving the robustness and adaptability of the trained network.

The baseline performance provided by the DeepLabv3+ model trained on each dataset offers a reference point for evaluating the effectiveness and enhancements achieved by our proposed methods.

### 4.4. Implementation Details

The pre-trained weights for SAM and SEEM were acquired from the official repositories of the projects (https://segment-anything.com/ (accessed on 23 July 2023) and https://github.com/UX-Decoder/Segment-Everything-Everywhere-All-At-Once (accessed on 23 July 2023)), hosted on the popular software development platform GitHub.

To evaluate the effectiveness of our proposed methods, checkpoints were calculated for every mask in the datasets utilized in this study. In this way, exactly the same checkpoint prompts were employed for each model to produce segmented masks, enabling a consistent and fair comparison across the different segmentation models.

### 4.5. Refinement Step Description

To further improve the segmentation results, we incorporated a final refinement step. This step involves combining the logit segmentation masks produced by the SAM model and the DeepLabv3+ model using a weighted-rule approach to obtain a final segmentation mask. For the sake of computation time, only for some datasets we also combine the logit masks produced by the SAM model and by the SOTA PVTv2 model.

The weighted rule combines the pixel-wise logit values from both models and applies a thresholding operation to generate a binary mask. The fusion process is formally described in Algorithm 2. We adjusted the weight of the segmentator model to 2. This modification helps balance the influence of the segmentator in the overall system.
**Algorithm 2** Combining continuous outputs of SAM and segmentator model.
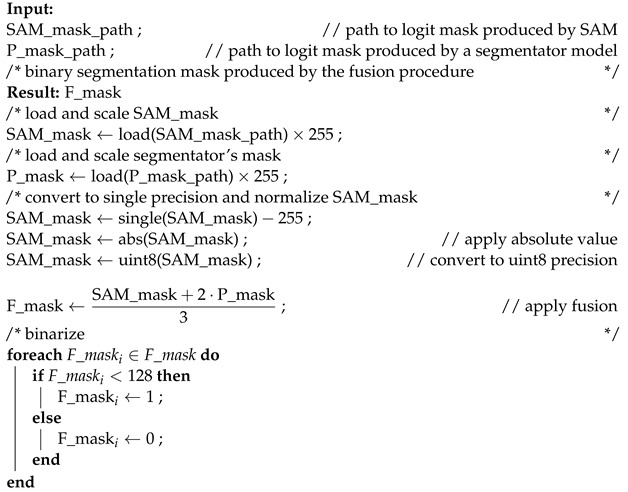


This refinement step is performed for several reasons.

- Combining complementary information: SAM and DeepLabv3+ have different strengths and weaknesses in capturing certain object details or handling specific image characteristics. By combining their logits through the weighted rule, we can leverage the complementary information captured by each model. This can lead to a more comprehensive representation of the object boundaries and semantic regions, resulting in a more accurate final segmentation.
- Noise reduction and consensus: the weighted-rule approach helps reduce the impact of noise or uncertainties in individual model predictions. By combining the logits, the noise or errors inherent in one model’s prediction may be offset or diminished by the other model’s more accurate predictions. This consensus-based aggregation can effectively filter out noisy predictions.
- Addressing model biases: different models can exhibit biases or tendencies in their predictions due to architectural differences, training data biases, or inherent limitations. The refinement step enables the combination of predictions from multiple models, mitigating the impact of any model biases and enhancing the overall robustness of the segmentation.
- Enhanced object boundary localization: the weighted rule of logits can help improve the localization and delineation of object boundaries. As the logits from both models contribute to the final segmentation, the refinement step tends to emphasize areas of high consensus, resulting in sharper and more accurate object boundaries. This can be particularly beneficial in cases where individual models may struggle with precise boundary detection.

It is important to note that to make the outputs of SAM and the other segmentators compatible, we scale them all to the same intensity range (0 to 255), making them suitable for direct comparison and further analysis. After scaling, we save them as gray levels and in .jpg format, for the sake of storage space. Another implementation detail of the refinement step is the inversion of SAM output masks values. SAM considers values near zero as background and values near 255 as objects, while our DeepLabv3+/PVTv2 code follows the opposite convention. To ensure compatibility between the models, we invert the SAM mask values.

## 5. Results and Discussion

### 5.1. Results

In this section we report and briefly analyze the results of the experiments, reported in Table 2 and Table 3. First, from the first group of experiments (Table 2, where we used the CAMO, Portrait, Locust-mini, and VinDr-RibCXR datasets) we noticed that the scores obtained by SEEM with all our checkpoint prompting strategies are lower than those obtained from SAM, or, at best, comparable. The only exception is the Dice score on the VinDr-RibCXR dataset, where SEEM overtakes SAM ViT-H by a small margin. However, the score remains markedly lower than the baseline. All in all, we can say that SEEM is less promising than SAM, at least with the prompting strategies and datasets we considered. This is the reason why SEEM was not included in the second group of experiments.

On average over all datasets, neither SAM (SAM ViT-L, SAM ViT-H) nor SEEM outperform DeepLabv3+, regardless of the prompts we provided. Not surprisingly, SEEM is worse on average than both SAM ViT-L and SAM ViT-H.

**Figure 3:**
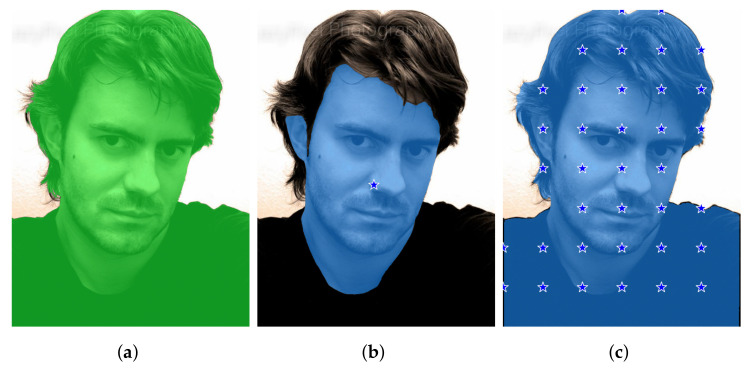
An example from the Portrait dataset demonstrating method A can fail to provide SAM with a prompt that is strong enough. (**a**) Ground truth. (**b**) Output of SAM with prompt extracted from the DeepLabv3+ mask by method A: a single checkpoint on the nose results in a segmentation output that can be considered semantically valid, but does not capture what was intended. (**c**) Output of SAM with prompt extracted from the DeepLabv3+ mask by method D (b=100, no mask erosion): a higher number of checkpoints pushes SAM to provide the intended segmentation mask.

The A, B, and C prompt generation methods are never the top performers, except for SAM ViT-L on the VinDr-RibCXR dataset. One reason for this fact is shown in Figure 3. The reason for including methods A to C in this paper is to document our experiments and discuss their points of failure.

From the results of the first group of experiments, no single variation of method D consistently provides the best performance. Sometimes (e.g., with the CAMO dataset) a low value of the sampling step *b* works best. Sometimes (e.g., with the Portrait dataset) a higher value of *b*, which produces fewer checkpoints, is beneficial. Sometimes the best results are obtained with mask erosion and sometimes not, albeit in these cases the difference is smaller and, in some of them, comparable with measurement noise. If a single value of the parameters must be chosen, then b=50 and no mask erosion provide good results on average. This is the value we adopt in the remaining experiments (Table 3, Table 4, Table 5 and Table 6). SAM overcomes DeepLabv3+ on the CAMO (Table 6) and COCO_animals (Table 3) datasets, performs similarly on the Portrait (Table 2) and Butterfly (Table 4) datasets, and is significantly worse than the baseline on the Locust-mini, VinDr-RibCXR (Table 2), and SKIN (Table 5) datasets. This is basically true for both the ViT-L and ViT-H models and for all the methods considered (oracle, DLV3+, PVTv2, and, on Butterfly, the SOTA ensemble introduced in [24]). Interestingly, in the COCO_animals dataset, SAMⓦDLV3+ significantly improved the baseline results, although the provided ground truth masks are not always accurate. This is why we also provided results in which the binarization of the DeepLabv3+ output mask, as described in Section 2, utilizes a stricter margin, specifically 32 instead of 128. By depending on a more robust response area of the source mask, it becomes more likely to exclude outlier checkpoints that could lead to incorrect SAM predictions. Subsequently, we integrate these results with the original DeepLabv3+ output logit mask, resulting in further enhancements in line with our expectations as shown in Table 3. Overall, remarkable results are obtained when using the fusion process described in Section 4.5 and Algorithm 2, for both the settings SAMⓦDLV3+ and SAMⓦPVTv2, where we always use the SAM ViT-H variant: in most datasets, the fusion method overcomes the corresponding baseline methods DLV3+ and PVTv2, sometimes by a large margin.

In particular, the strongest results of this article are obtained with the CAMO dataset, as highlighted in Table 6. The prompts we extract from DeepLabv3+ masks with method D allow SAM to outperform DeepLabv3+, that is, to provide a better mask than the one of DeepLabv3+ itself. Most importantly, the fusion between SAM and the ensemble of PVTv2 outperforms the ensemble of PVTv2, which is a current SOTA segmentation approach. For comparison, we report the performance of Explicit Visual Prompting v2 (EVPv2) [38], which has the best SOTA metrics that are available on the famous benchmark dataset sharing platform Papers With Code (https://paperswithcode.com/sota/camouflaged-object-segmentation-on-camo (accessed on 01 July 2023)). In other words, the fusion between PVTv2 and SAM-based segmentators becomes, to the best of our knowledge, the new SOTA on the CAMO dataset. Significant results are also obtained with the SKIN and Butterfly datasets. On SKIN (Table 5), the ensembles (fusions between SAM and DeepLabv3+ or PVTv2) beat the baselines (masks calculated by DeepLabv3+ or PVTv2) on many of the subsets of images that comprise the dataset. On Butterfly (Table 4), the ensemble of SAM with the SOTA model mostly overcomes the SOTA model itself, although by a small margin; SOTA is an ensemble of PVTv2 and CNN-based segmentators. On COCO_animals (Table 3), the already good results obtained by SAM are further improved when merged with DeepLabv3+. Moreover, we observe that changing the fusion segmentator’s mask weight (2) reported in Algorithm 2 to different values, i.e., increasing it (e.g., to 3), brings slightly better results in both IoU and Dice scores. We postpone this observation as a future work to investigate the tuning of the fusion weight.

### 5.2. Discussion

In this section, we supplement the summary of the results provided in Section 5.1 with some general remarks about the strengths and failure modes that we encountered in our experiments. The analysis includes a collection of figures that illustrate our assertions, offering a visual demonstration of the capabilities and drawbacks of the proposed prompting methods and the zero-shot segmentators we consider.

**Figure 4:**
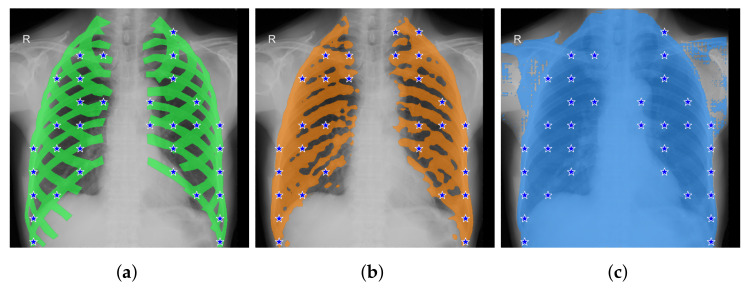
A typical failure mode of SAM on the VinDr-RibCXR dataset: basically, SAM does not capture any anatomical structure. (**a**) Ground truth and corresponding checkpoints extracted by method D (oracle method, b=50, no mask erosion). (**b**) Mask from DeepLabv3+ and corresponding checkpoints extracted by method D (b=50, no mask erosion). (**c**) The output of SAM-DLV3+ when prompted with oracular checkpoints from (**a**). It is apparent that the mask is much worse than that provided by DeepLabv3+.

The oracle method, included in the first group of experiments, provides a significant performance boost on the CAMO and Locust-mini datasets, but not on the Portrait and VinDr-RibCXR datasets. This is true for both the SAM and SEEM models. We think the reason may be the same for Portrait and VinDr-RibCXR. The performance on the former dataset is so good that the improvement in prompting with the oracle method produces negligible effects. The performance on the latter dataset is so poor, that is, the models are so inadequate to segment anatomical structures such as ribs (Figure 4), that changes in prompting do not make a difference. At present, it is unclear if the issue is simply due to the lack of medical images in the training set of the models, or to the very structures of the models.

**Figure 5:**
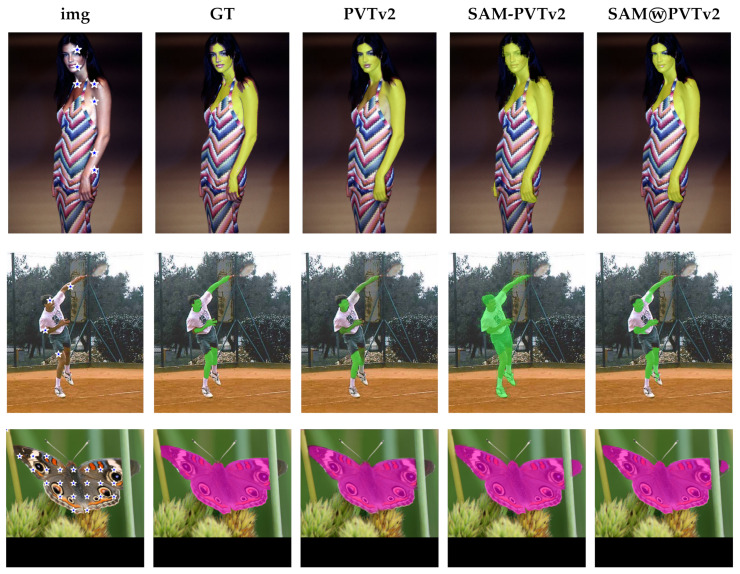
Examples from the SKIN and Butterfly datasets. Arranged from left to right are: the source image (img), the ground truth mask (GT), the mask predicted by PVTv2 (PVTv2), the binary mask produced by SAM using checkpoints from the PVTv2 mask (SAM-PVTv2), and the binary mask obtained by fusing SAM and PVTv2 masks (SAMⓦPVTv2). From top to bottom: examples from SKIN CMQ, SKIN UC, and Butterfly (Folder 3).

**Figure 6:**
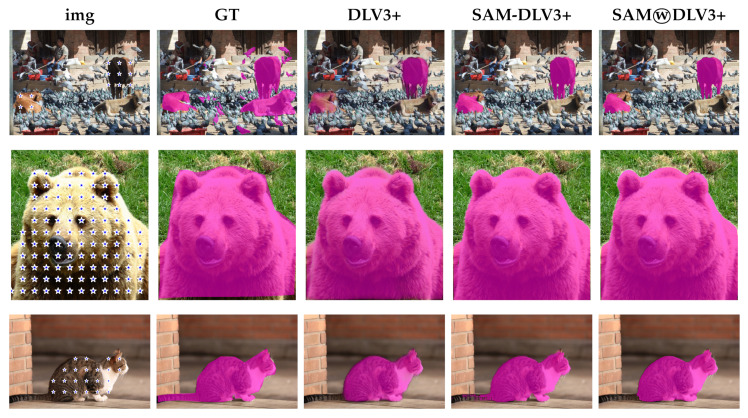
Examples from the COCO 2017 dataset. Arranged from left to right are: the source image (img), the ground truth mask (GT), the mask predicted by DeepLabv3+ (DLV3+), the binary mask generated by SAM using checkpoints from the DeepLabv3+ mask (SAM-DLV3+), and the binary mask obtained by fusing SAM and DeepLabv3+ masks (SAMⓦDLV3+). The first row illustrates one of the many cases in which the ground truth exhibits lack of details, that is, not all birds are segmented and even the masks for big animals are far from perfect. The second row depicts a good catch of SAM: the predicted segmentation mask is much more detailed than the ground truth. In the third row, both DeepLabv3+ and SAM perform a very detailed segmentation but miss the cat’s tail.

**Figure 7:**
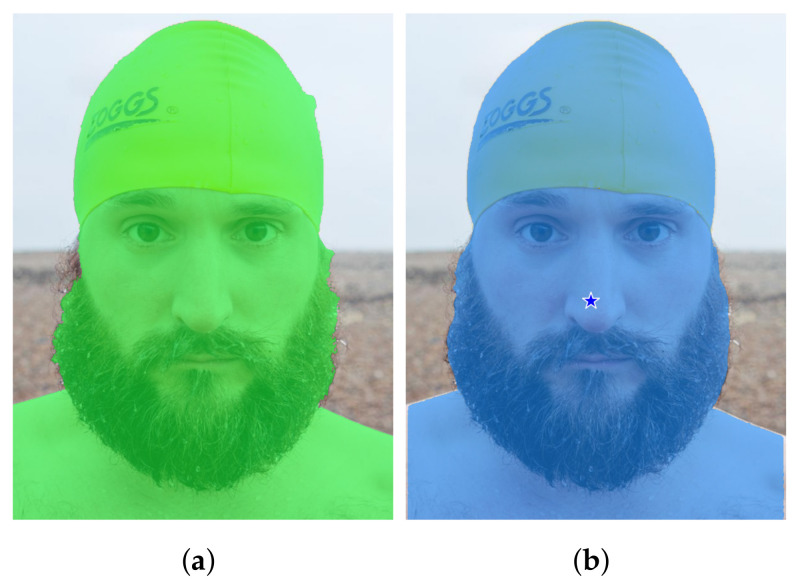
An example from the Portrait dataset that shows method A operating under the same conditions described in Figure 3, but providing a strong enough hint. (**a**) Ground truth. (**b**) Output of SAM-DLV3+ with prompt extracted from the DeepLabv3+ mask by method A: in this case, a single checkpoint on the nose results in the correct segmentation output by SAM-DLV3+. Indeed, the mask provided by SAM-DLV3+ is better than the ground truth in the beard region.

According to the results in Table 2, the added complexity of the SAM ViT-H model compared to the SAM ViT-L model does not make a radical difference in the Portrait dataset. As a matter of fact, the smaller model performs slightly better than the larger one. We believe that, similarly to the case we previously discussed, performance on the Portrait dataset is already so high with ViT-L that a wall has been hit. It is difficult to overcome such a wall by simply increasing the complexity of the model. However, if we look at the other three datasets, we observe that SAM ViT-H performs significantly better than SAM ViT-L. It is important to note that the combination (FUSION method in Table 2) of the logit masks provided by SAM and the segmentation model (either DeepLabv3+ or PVTv2) used to extract checkpoints consistently exceed the performance achieved by SAM alone in almost all datasets. Only in the CAMO dataset with checkpoints extracted by DeepLabv3+ the performance of SAM is slightly better than the fusion SAMⓦDLV3+, while in the same dataset the SAMⓦPVTv2 fusion obtains results which, to the best of our knowledge, represent the new state-of-the-art for this dataset. In other datasets, the improvement of the SAMⓦDLV3+ combination is substantial with respect to SAM, e.g., in VinDr-RibCXR, Locust-mini, and Butterfly datasets. A final observation concerns the quality of the ground truth of the datasets, which sometimes limits the possibility of effective performance improvement but, on the other hand, provides further motivation for the use in the segmentation pipelines of zero-shot segmentators such as SAM and SEEM. Indeed, we found images in which the ground truth is wrong. We encountered this issue in the SKIN (Figure 5, first row of images: one of the two hands is missing from the ground truth), COCO (Figure 6) and Portrait (see, e.g., Figure 7a, swimming cap and beard) datasets.

**Figure 8:**
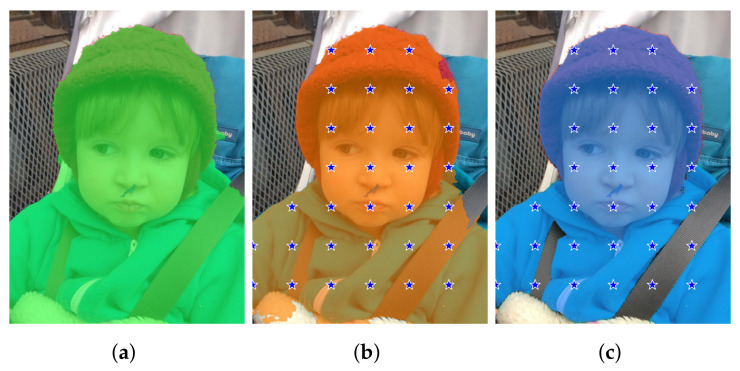
An example from the Portrait dataset where the output of SAM-DLV3+, with suitable prompting obtained from the DeepLabv3+ mask, is arguably better than the DeepLabv3+ mask itself. (**a**) Ground truth. (**b**) Mask from DeepLabv3+ and checkpoints extracted from such mask by method D (b=100, no mask erosion). (**c**) Mask provided by SAM-DLV3+. It can be seen that SAM-DLV3+ ignores the belts, albeit hinted to include them, and the blanket. It can be argued that this choice is semantically better than the output of DeepLabv3+ and the ground truth, where the mask includes objects that are not part of the person.

In other images, the ground truth is, at least, semantically questionable (Figure 8a, belts and blanket). In these cases, SAM or SEEM typically provides a mask that is more accurate than the ground truth (see, e.g., Figure 7b and Figure 8c, and the aforementioned Figure 5 and Figure 6). The net result is that these images erroneously lower the IoU and Dice scores for SAM and SEEM.

Finally, we investigate the option to fuse two specialized models such as DLV3+ and PVTv2 to assess the effectiveness of our insights. In Table 7, we compare the standalone DLV3+ and PVTv2 methods, two different ensembles of them (using the average rule and weighted rule, with PVTv2 weighed twice as much), and our method, which can be ensembled with either DLV3+ or PVTv2. This evaluation is carried out on CAMO, SKIN and Butterfly datasets. It is important to note that PVTv2 is SOTA in these datasets. As shown in the table, SAMⓦPVTv2 exhibits better performance than the other models on CAMO and Butterfly, with occasional outperformance by DLV3+ⓐPVTv2 or DLV3+ⓦPVTv2 in some SKIN subsets. This outperformance comes at the cost of a double training, i.e., two specialized models must be trained on the target dataset. In our method, the fusion of SAM with a specialized model comes at no additional cost.

In Table 8 we report summary results obtained by averaging various subsets within each dataset used in this paper. The ensemble of SAM with DLV3+ or PVTv2 is consistently the best performing, except for the VinDr-RibCXR and SKIN datasets. These findings demonstrate the effectiveness of the SAM method in enhancing the accuracy of other predictors.

## 6. Conclusions

**Figure 9:**
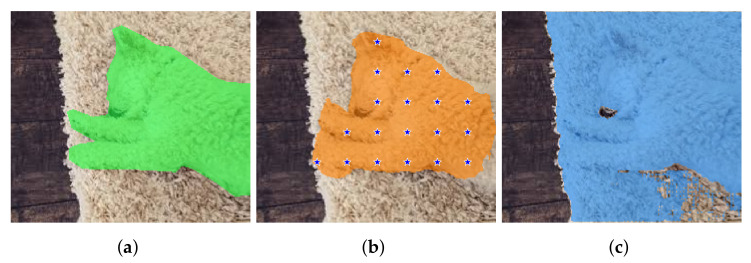
A failure mode of SAM-DLV3+ on the CAMO dataset: despite strong prompting, SAM-DLV3+ fails to segment a common pet. (**a**) Ground truth. (**b**) Mask from DeepLabv3+ and corresponding checkpoints extracted by method D (b=30, no mask erosion). (**c**) The output of SAM-DLV3+ when prompted with the aforementioned checkpoints (not shown).

**Figure 10:**
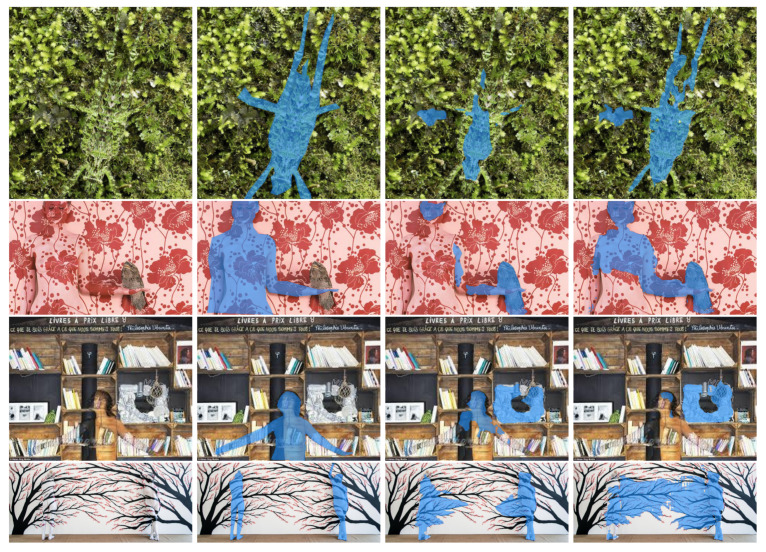
Further examples from the CAMO dataset. Arranged from left to right are: the source images, the ground truth masks, the binary masks obtained by the PVTv2 method, the binary masks obtained by fusing the logit masks output by SAM when prompted with the aforementioned checkpoints (SAM-PVTv2). The first two rows demonstrate significant improvements in segmentation, while the last two rows illustrate instances where the fusion process did not yield the desired results.

**Figure 11:**
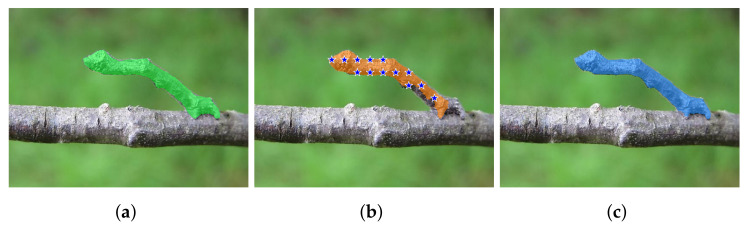
An example from the CAMO dataset where SAM provides a better mask than DeepLabv3+. (**a**) Ground truth. (**b**) Mask from DeepLabv3+ and corresponding checkpoints extracted by method D (b=30, no mask erosion). (**c**) The output of SAM-DLV3+ when prompted with the aforementioned checkpoints (not shown for clarity).

Our experiments demonstrate that exploiting a state of the art zero-shot segmentator as SAM alongside specialized segmentation models can lead to general segmentation improvements. We have also shown that a combination of segmentators at the logit level (i.e., SAMwDLV3+ or SAMwPVTv2) can lead to segmentation improvements over the original masks obtained by mainstream segmentators, even beating the SOTA in the CAMO and Butterfly datasets. Sometimes, the zero-shot segmentator is even resilient to errors in prompting caused by inaccuracies in the original mask (see, e.g., Figure 8). These findings highlight the potential of zero-shot segmentators such as SAM and SEEM to advance the state of the art for semantic segmentation. However, current zero-shot segmentators are no panacea. Sometimes, a similar prompt produces different results in different images (Figure 3b and Figure 7b). Sometimes, the output mask is not semantically acceptable despite what we already believe to be a strong prompt (Figure 9 and Figure 10). Sometimes, the results are bad on whole datasets: we encountered this issue with SKIN and VinDr-RibCXR. As discussed in Section 2.2, there is evidence that the accuracy of SAM in medical image segmentation tasks is not excellent. In any case, the failure modes we encountered in our experiments provide valuable insights for further analysis. To this aim, future work should consider a larger number of datasets, especially from additional domains, to determine when performance issues are domain-specific. Other prompting strategies must be investigated, chiefly those based on bounding boxes and text prompts. We remark that prompting SAM with text, although explored in [2], is currently inaccessible with the source code that is publicly available. Moreover, future work also need to investigate the tuning of the fusion weight parameter, in order to optimize the contributions of the segmentator’s mask and the SAM’s predicted mask. Of course, a long-term objective is to identify variations in the architecture of the model that make the final result more resilient to weak prompting. Variations include both the substitution of DeepLabv3+ and PVTv2 with other models and modifications to zero-shot segmentators (see Figure 11).

## Figures and Tables

**Table 1:** Description of the different skin datasets. ECU datasets are split into 2000 images for training and 2000 as a further test set.

Short Name	Complete Name	Num of Samples
CMQ	Compaq	4675
ECU	ECU Face and Skin Detection	2000
HGR	Hand Gesture Recognition	1558
MCG	MCG-skin	1000
Prat	Pratheepan	78
Sch	Schmugge dataset	845
SFA	SFA	1118
UC	UChile DB-skin	103
VMD	Human activity recognition	285
VT	VT-AAST	66

**Table 2:** Experimental results of different methods on four datasets using the Intersection over Union (IoU) and Dice similarity coefficient (Dice) as evaluation metrics. The methods compared in the table include variations of the ViT-H and ViT-L models, as well as the SEEM method. In the top the results of the DeepLabv3+ model (our baseline) and the SOTA method PVTv2 are reported. In the “Method” column, the number represents the grid sampling step size that we call *b* in Section 3.5. The FUSION method represents either SAMⓦDLV3+ or SAMⓦPVTv2. We always use the SAM ViT-H variant. The word “bm” denotes whether the masks from which the checkpoints were extracted were eroded (to avoid checkpoints too close to the borders) or not.

		CAMO						Portrait				Locust-Mini				VinDr-RibCXR			
		IoU			Dice			IoU		Dice		IoU		Dice		IoU		Dice	
Baseline (DLV3+)		60.63			71.75			97.01		98.46		74.34		83.01		63.48		77.57	
PVTv2-Ensemble		71.75			81.07														
		Oracle		DLV3+		PVTv2		Oracle		DLV3+		Oracle		DLV3+		Oracle		DLV3+	
Model	Method	IoU	Dice	IoU	Dice	IoU	Dice	IoU	Dice	IoU	Dice	IoU	Dice	IoU	Dice	IoU	Dice	IoU	Dice
SAM-ViT-L	A	51.26	60.42	47.96	58.36	49.95	59.97	57.31	67.96	55.50	66.49	48.43	59.56	35.45	46.15	**37.30**	**53.96**	**30.00**	**45.76**
	B	50.57	59.66	48.10	58.49	49.73	59.72	57.43	68.04	55.48	66.41	48.55	59.81	35.53	46.22	37.30	53.96	29.98	45.73
	C	44.30	53.50	44.06	54.53	44.21	53.89	55.24	65.20	52.28	62.57	36.50	45.77	34.10	44.36	31.43	47.45	28.83	44.41
	D 10	37.87	51.54	38.01	51.03	36.64	49.93	77.59	85.96	77.64	86.01	21.47	33.22	23.14	35.02	25.66	40.65	25.64	40.62
	D 30	62.75	73.61	58.81	69.45	59.31	69.95	91.99	95.55	91.87	95.47	48.05	59.62	51.93	63.56	26.77	42.06	26.97	42.34
	D 50	67.29	76.93	59.84	69.62	62.89	72.49	95.97	97.91	95.72	97.77	56.46	66.97	59.48	69.88	28.24	43.76	27.96	43.51
	D 100	61.74	69.92	53.23	61.73	57.00	65.52	**96.31**	**98.09**	**96.16**	98.00	50.67	59.70	51.89	60.66	29.99	45.80	29.62	45.40
	D 10 bm	44.42	57.99	41.11	53.92	40.29	53.31	78.09	86.34	78.15	86.39	35.42	48.34	27.33	39.73	26.57	41.82	25.75	40.77
	D 30 bm	67.17	77.44	60.13	70.47	60.98	71.25	92.41	95.81	92.23	95.71	59.11	70.08	54.32	65.73	29.26	45.01	26.96	42.32
	D 50 bm	**69.40**	**78.71**	**60.89**	**70.73**	**63.02**	**72.67**	96.01	97.93	95.79	97.81	**61.89**	**72.28**	**59.74**	**70.21**	30.19	46.04	28.01	43.58
	D 100 bm	62.80	71.67	54.05	62.91	58.01	66.98	96.26	98.06	96.16	**98.01**	53.09	62.34	52.69	61.77	31.86	47.82	29.39	45.03
SAM-ViT-H	A	50.87	59.77	50.56	60.62	52.49	62.07	54.24	64.63	51.42	62.35	48.83	60.11	40.09	51.05	33.78	50.10	31.21	47.23
	B	50.55	59.44	50.57	60.61	52.42	61.99	54.33	64.79	51.75	62.67	48.91	60.21	40.89	51.80	33.76	50.08	31.14	47.14
	C	45.68	54.43	47.32	57.34	49.14	58.63	48.83	59.31	48.01	58.49	42.46	52.03	34.21	44.30	29.23	44.76	30.37	46.24
	D 10	60.80	72.85	53.53	66.36	54.86	67.40	77.19	86.11	76.91	85.88	40.50	55.19	40.18	54.47	28.94	44.73	29.01	44.80
	D 30	**78.49**	86.37	**65.44**	**75.46**	**70.19**	**79.28**	92.73	96.14	92.24	95.85	70.81	80.78	68.64	78.43	37.79	54.65	38.17	55.07
	D 50	77.93	85.81	63.46	73.00	69.83	78.49	**96.38**	**98.14**	**95.98**	**97.92**	67.85	77.92	**68.89**	**78.69**	37.89	54.61	**38.67**	**55.48**
	D 100	66.65	73.95	55.45	63.55	59.97	67.94	95.01	97.40	94.76	97.26	51.64	60.55	53.54	62.14	31.22	47.18	31.37	47.41
	D 10 bm	65.27	76.57	55.01	67.53	56.71	68.86	77.31	86.18	77.00	85.93	57.04	70.34	45.88	59.23	35.79	52.51	29.46	45.36
	D 30 bm	78.12	**86.46**	64.95	75.18	69.81	79.10	92.96	96.27	92.54	96.02	**71.51**	**81.53**	68.67	78.58	**39.41**	**56.23**	38.16	55.05
	D 50 bm	76.02	84.26	63.37	73.21	69.00	77.98	96.38	98.14	95.98	97.92	68.05	78.34	68.53	78.46	35.53	51.95	38.45	55.22
	D 100 bm	67.02	75.45	56.22	64.67	61.75	70.10	94.99	97.38	94.74	97.25	53.26	62.54	54.56	63.44	31.13	46.99	31.28	47.28
SEEM	A	48.24	55.46	38.58	44.58	38.12	44.52	93.52	95.73	92.94	95.23	39.20	47.81	35.87	43.31	32.13	48.42	32.12	48.41
	B	48.24	55.46	38.37	44.38	37.82	44.21	93.52	95.73	92.94	95.23	39.20	47.81	35.87	43.31	32.13	48.42	32.12	48.41
	C	44.64	51.09	41.65	47.76	33.97	39.67	92.31	94.45	89.84	92.14	32.98	40.61	26.25	32.73	31.58	47.78	31.90	48.18
	D 10	57.77	65.56	53.82	61.56	45.39	52.46	95.90	97.88	95.87	97.86	**63.93**	**72.13**	58.21	65.93	32.15	48.46	32.05	48.35
	D 30	57.18	64.76	53.37	61.08	52.74	60.16	95.89	97.87	95.86	97.86	61.97	69.96	**59.54**	**67.14**	32.13	48.43	32.12	**48.42**
	D 50	55.09	62.23	51.64	58.74	50.91	58.10	95.85	97.85	95.84	97.84	59.30	67.12	58.35	65.94	31.98	48.26	32.05	48.33
	D 100	51.92	58.68	49.14	55.89	50.38	57.16	95.80	97.83	95.80	97.82	47.42	55.07	43.45	50.74	31.79	48.03	31.83	48.08
	D 10 bm	**58.89**	**66.79**	**54.26**	**62.11**	**52.97**	**60.75**	**95.92**	97.89	95.91	97.88	62.39	70.80	57.33	65.59	**32.17**	**48.48**	**32.12**	48.42
	D 30 bm	57.57	65.25	53.27	61.03	52.87	60.29	95.92	**97.89**	**95.91**	**97.88**	60.79	68.74	58.10	65.68	32.07	48.35	32.11	48.42
	D 50 bm	55.11	62.38	51.62	58.65	51.55	58.77	95.89	97.87	95.89	97.87	58.16	66.20	57.05	64.71	31.94	48.19	32.05	48.33
	D 100 bm	51.78	58.62	48.83	55.53	49.86	56.67	95.81	97.83	95.79	97.82	47.67	55.43	43.82	51.14	31.71	47.94	31.82	48.06
FUSION	D 30	-	-	**63.87**	**74.41**	73.31	**82.02**	-	-	97.13	98.53	-	-	75.99	84.21	-	-	**60.96**	**75.64**
	D 50	-	-	62.95	73.36	**73.46**	82.02	-	-	**97.18**	**98.55**	-	-	75.74	84.07	-	-	60.65	75.41
	D 30 bm	-	-	63.48	74.18	73.13	81.92	-	-	97.14	98.53	-	-	**76.01**	**84.23**	-	-	60.95	75.63
	D 50 bm	-	-	63.03	73.54	73.27	81.94	-	-	97.17	98.55	-	-	75.74	84.07	-	-	60.67	75.43

**Table 3:** IoU and Dice results on the COCO dataset. A metric is on each row, while the columns report the metric value of the different methods. From left to right: DeepLabv3+, SAM ViT-H with prompts obtained from DeepLabv3+ masks (method D, b=50, no mask erosion), fusion of the masks just mentioned with DeepLabv3+ masks, DeepLabv3+ (here indicated with the asterisk, *DeepLabv3+, or in the table with *DLV3+) masks but with a different binarization threshold, i.e., 32 instead of 128, SAM ViT-H with prompts obtained from *DeepLabv3+, fusion of the masks just mentioned with *DeepLabv3+ masks. The up-arrow ↑ means that higher is better. *b* is the grid sampling step size.

COCO_Animals	DLV3+	SAM-DLV3+	SAM ⓦ DLV3+	*DLV3+	SAM-*DLV3+	SAM ⓦ *DLV3+
**IoU ↑**	66.04	67.45	**69.15**	54.61	65.59	68.24
**Dice ↑**	75.93	75.19	**77.93**	66.53	73.17	77.26

**Table 4:** IoU and Dice results on the Butterfly dataset. A subset of the dataset is on each row, while the columns report the results of the different methods. From left to right: DeepLabv3+, SAM ViT-H with prompts obtained from DeepLabv3+ masks (method D, b=50, no mask erosion), fusion of the masks just mentioned with DeepLabv3+ masks, SOTA [24], SAM ViT-H with prompts obtained from SOTA masks (method D, b=50, no mask erosion), fusion of the masks just mentioned with SOTA masks. The up-arrow ↑ means that higher is better. *b* is the grid sampling step size.

		DLV3+	SAM-DLV3+	SAM ⓦ DLV3+	SOTA	SAM-SOTA	SAM ⓦ SOTA
**IoU**↑	Fold_1	95.57	92.57	96.11	**96.95**	93.85	96.92
	Fold_2	95.68	93.28	96.13	97.13	93.52	**97.15**
	Fold_3	95.26	92.81	95.71	97.00	93.92	**97.04**
	Fold_4	95.37	92.55	96.07	96.90	94.09	**96.92**
**Dice**↑	Fold_1	97.72	95.80	98.01	**98.44**	96.73	98.43
	Fold_2	97.78	96.44	98.01	98.54	96.57	**98.55**
	Fold_3	97.53	96.13	97.77	98.47	96.81	**98.49**
	Fold_4	97.62	95.75	97.98	98.42	96.91	**98.43**

**Table 5:** IoU and Dice results on the SKIN dataset. A subset of the dataset is on each row, while the columns report the results of the different methods. From left to right: DeepLabv3+, SAM ViT-H with prompts obtained from DeepLabv3+ masks (method D, b=50, no mask erosion), fusion of the masks just mentioned with DeepLabv3+ masks, PVTv2, SAM ViT-H with prompts obtained from PVTv2 masks (method D, b=50, no mask erosion), fusion of the masks just mentioned with PVTv2 masks. The up-arrow ↑ means that higher is better. *b* is the grid sampling step size.

		DLV3+	SAM-DLV3+	SAM ⓦ DLV3+	PVTv2	SAM-PVTv2	SAM ⓦ PVTv2
**IoU**↑	CMQ	73.58	68.22	74.37	76.62	68.09	**76.75**
	ECU	90.34	86.26	90.31	91.30	86.38	**91.34**
	HGR	94.32	94.42	**94.73**	94.34	94.35	94.64
	MCG	79.81	79.34	80.67	80.74	79.70	**81.33**
	Prat	85.75	73.81	86.63	86.16	74.85	**87.14**
	Sch	63.72	52.02	63.07	**66.15**	53.68	65.51
	SFA	90.72	91.58	91.56	91.25	91.72	**91.80**
	UC	84.71	67.00	85.67	**87.49**	62.89	82.99
	VMD	59.49	33.51	**60.86**	59.78	33.33	59.31
	VT	65.96	47.28	69.48	74.59	47.65	**76.64**
**Dice**↑	CMQ	84.78	81.11	85.30	86.77	81.01	86.85
	ECU	94.92	92.63	94.91	95.45	92.69	**95.48**
	HGR	97.08	97.13	**97.29**	97.09	97.09	97.25
	MCG	88.77	88.48	89.30	89.34	88.70	**89.71**
	Prat	92.33	84.93	92.84	92.57	85.62	**93.13**
	Sch	77.84	68.44	77.35	**79.63**	69.86	79.16
	SFA	95.13	95.61	95.59	95.42	95.68	**95.73**
	UC	91.72	80.24	92.28	**93.33**	77.22	90.70
	VMD	74.60	50.20	**75.67**	74.83	50.00	74.46
	VT	79.49	64.21	81.99	85.45	64.54	**86.78**

**Table 6:** Complete results on the CAMO dataset. Line 1: DeepLabv3+. Line 2: PVTv2. Line 3: EVPv2 (current State of the Art method on CAMO dataset). Line 4: SAM with prompts obtained from DeepLabv3+ masks (method D, b=50, no mask erosion). Line 5: fusion of the masks just mentioned with DeepLabv3+ masks. Line 6: SAM with prompts obtained from PVTv2 masks (method D, b=50, no mask erosion). Line 7: fusion of the masks just mentioned with PVTv2 masks. ↑ means that higher is better, ↓ means that lower is better. The number represents the grid sampling step size that we call *b* in the description Section 3.5.

	IoU ↑	Dice ↑	MAE ↓	F-Score ↑	E-Measure ↑
DLV3+	60.63	71.75	8.39	75.57	83.04
PVTv2-ensemble	71.75	81.07	5.74	82.46	89.96
EVPv2 (current SOTA)	-	-	5.80	78.60	89.90
SAM-DLV3+	63.46	73.00	9.78	74.25	81.90
SAM ⓦ DLV3+	62.95	73.36	8.02	76.92	83.49
SAM-PVTv2	69.83	78.49	7.67	78.96	86.21
SAM ⓦ PVTv2	**73.46**	**82.02**	**5.45**	**83.56**	**90.00**

**Table 7:** IoU and Dice results on the datasets that were tested using PVTv2 model, from the top: CAMO, SKIN and Butterfly. From the third column, left to right: DeepLabv3+, PVTv2, ensemble between DeepLabv3+ and PVTv2 using the average rule, ensemble between DeepLabv3+ and PVTv2 using the weighted rule (the latter weighed twice as much), ensemble of SAM ViT-H with prompts obtained from DeepLabv3+ (method D, b=50, no mask erosion) and DeepLabv3+ masks, ensemble of SAM ViT-H with prompts obtained from PVTv2 (method D, b=50, no mask erosion) and PVTv2 masks. The up-arrow ↑ means that higher is better. *b* is the grid sampling step size.

Dataset	Metric	DLV3+	PVTv2	DLV3+ ⓐ PVTv2	DLV3+ ⓦ PVTv2	SAM ⓦ DLV3+	SAM ⓦ PVTv2
**CAMO**	**IoU ↑**	60.63	71.75	69.32	71.23	62.95	**73.46**
	**Dice ↑**	71.75	81.07	79.16	80.60	73.36	**82.02**
**CMQ**	**IoU ↑**	73.58	76.62	77.09	**77.30**	74.37	76.75
	**Dice ↑**	84.78	86.77	87.07	**87.20**	85.30	86.85
**ECU**	**IoU ↑**	90.34	91.30	**91.55**	91.54	90.31	91.34
	**Dice ↑**	94.92	95.45	**95.59**	95.58	94.91	95.48
**HGR**	**IoU ↑**	94.32	94.34	94.66	94.55	**94.73**	94.64
	**Dice ↑**	97.08	97.09	97.26	97.20	**97.29**	97.25
**MCG**	**IoU ↑**	79.81	80.74	80.85	80.85	80.67	**81.33**
	**Dice ↑**	88.77	89.34	89.41	89.41	89.30	**89.71**
**Prat**	**IoU ↑**	85.75	86.16	86.80	86.66	86.63	**87.14**
	**Dice ↑**	92.33	92.57	92.93	92.85	92.84	93.13
**Sch**	**IoU ↑**	63.72	66.15	66.88	**67.27**	63.07	65.51
	**Dice ↑**	77.84	79.63	80.15	**80.43**	77.35	79.16
**SFA**	**IoU ↑**	90.72	91.25	91.55	91.47	91.56	**91.80**
	**Dice ↑**	95.13	95.42	95.59	95.55	95.59	**95.73**
**UC**	**IoU ↑**	84.71	87.49	88.88	**88.92**	85.67	82.99
	**Dice ↑**	91.72	93.33	94.11	**94.13**	92.28	90.70
**VMD**	**IoU ↑**	59.49	59.78	**63.51**	63.41	60.86	59.31
	**Dice ↑**	74.60	74.83	**77.68**	77.61	75.67	74.46
**VT**	**IoU ↑**	65.96	74.59	71.73	73.04	69.48	**76.64**
	**Dice ↑**	79.49	85.45	83.54	84.42	81.99	**86.78**
Fold_1	**IoU ↑**	95.57	96.95	96.83	**96.94**	96.11	96.92
	**Dice ↑**	97.72	98.44	98.38	**98.44**	98.01	98.43
Fold_2	**IoU ↑**	95.68	97.13	97.00	97.13	96.13	**97.15**
	**Dice ↑**	97.78	98.54	98.47	98.54	98.01	**98.55**
Fold_3	**IoU ↑**	95.26	97.00	96.67	96.86	95.71	**97.04**
	**Dice ↑**	97.53	98.47	98.29	98.39	97.77	**98.49**
Fold_4	**IoU ↑**	95.37	96.90	96.77	96.90	96.14	**96.95**
	**Dice ↑**	97.62	98.42	98.35	98.42	97.98	**98.43**

**Table 8:** IoU and Dice summary results computed by averaging various subsets within each dataset used in this paper. Please refer to Table 7 for the legend explaining the order from left to right.

Dataset	Metric	DLV3+	PVTv2	DLV3+ ⓐ PVTv2	DLV3+ ⓦ PVTv2	SAM ⓦ DLV3+	SAM ⓦ PVTv2
**CAMO**	**IoU ↑**	60.63	71.75	69.32	71.23	62.95	**73.46**
	**Dice ↑**	71.75	81.07	79.16	80.60	73.36	**82.02**
**Portrait**	**IoU ↑**	97.01	-	-	-	**97.18**	-
	**Dice ↑**	98.46	-	-	-	**98.55**	-
**Locust-mini**	**IoU ↑**	74.34	-	-	-	**75.74**	-
	**Dice ↑**	83.01	-	-	-	**84.07**	-
**VinDr-RibCXR**	**IoU ↑**	**63.48**	-	-	-	60.65	-
	**Dice ↑**	**77.57**	-	-	-	75.41	-
**SKIN**	**IoU ↑**	78.84	80.84	81.35	**81.50**	79.73	80.74
	**Dice ↑**	87.67	88.99	89.33	**89.44**	88.25	88.92
**Butterfly**	**IoU ↑**	95.47	97.00	96.82	96.96	96.02	**97.02**
	**Dice ↑**	97.66	98.47	98.37	98.45	97.94	**98.48**
**COCO animals**	**IoU ↑**	66.04	-	-	-	**69.15**	-
	**Dice ↑**	75.93	-	-	-	**77.93**	-

## Data Availability

All the resources required to replicate our experiments are available at https://github.com/LorisNanni (accessed on 22 July 2023).
